# Cytoskeleton—a crucial key in host cell for coronavirus infection

**DOI:** 10.1093/jmcb/mjaa042

**Published:** 2020-07-27

**Authors:** Zeyu Wen, Yue Zhang, Zhekai Lin, Kun Shi, Yaming Jiu

**Affiliations:** 1 The Center for Microbes, Development and Health, Key Laboratory of Molecular Virology and Immunology, Institut Pasteur of Shanghai, Chinese Academy of Sciences, Shanghai 200031, China; 2 University of Chinese Academy of Sciences, Beijing 100049, China; 3 Department of Gynecology and Obstetrics, Guangzhou Women and Children’s Medical Center, Guangzhou Medical University, Guangzhou 510623, China

**Keywords:** coronavirus, host cytoskeleton, actin filaments, microtubules, intermediate filaments, pathology

## Abstract

The emerging coronavirus (CoV) pandemic is threatening the public health all over the world. Cytoskeleton is an intricate network involved in controlling cell shape, cargo transport, signal transduction, and cell division. Infection biology studies have illuminated essential roles for cytoskeleton in mediating the outcome of host‒virus interactions. In this review, we discuss the dynamic interactions between actin filaments, microtubules, intermediate filaments, and CoVs. In one round of viral life cycle, CoVs surf along filopodia on the host membrane to the entry sites, utilize specific intermediate filament protein as co-receptor to enter target cells, hijack microtubules for transportation to replication and assembly sites, and promote actin filaments polymerization to provide forces for egress. During CoV infection, disruption of host cytoskeleton homeostasis and modification state is tightly connected to pathological processes, such as defective cytokinesis, demyelinating, cilia loss, and neuron necrosis. There are increasing mechanistic studies on cytoskeleton upon CoV infection, such as viral protein‒cytoskeleton interaction, changes in the expression and post-translation modification, related signaling pathways, and incorporation with other host factors. Collectively, these insights provide new concepts for fundamental virology and the control of CoV infection.

## Introduction

Coronaviruses (CoVs) are enveloped viruses with a positive-sense, single-stranded RNA genome and belong to the *Coronaviridae* family, *Nidovirales* order. The genome of CoVs encodes replicase‒transcriptase polyprotein and four structural proteins, i.e. spike (S), envelope (E), membrane (M), and nucleocapsid (N). The most prominent feature of CoVs is the club-shape spike projections emanating from the virion surface, which is responsible for the interaction between virus and cellular receptors (Snijder et al., 2003; Fehr and Perlman, 2015). There are distinct entry patterns for CoVs, including plasma membrane fusion, phagocytosis, micropinocytosis, and clathrin-mediated or clathrin-independent endocytosis (Kumari et al., 2010; Mayor et al., 2014; Fehr and Perlman, 2015). After entry, the viral replicase gene translates into polyprotein that can self cleaves to form nonstructural proteins, and subsequently assemble into replicase‒transcriptase complex (RTC) to create a suitable environment for RNA synthesis (Fehr and Perlman, 2015). Following replication and subgenomic RNA synthesis, the viral structural proteins traffic to the endoplasmic reticulum (ER)‒Golgi intermediate compartment (ERGIC), and then encapsulate viral genomes and form mature virions via budding (Tooze et al., 1984; Krijnse-Locker et al., 1994). Finally, virions are transported to the cell surface and released through exocytosis (Fehr and Perlman, 2015).

**Figure 1:**
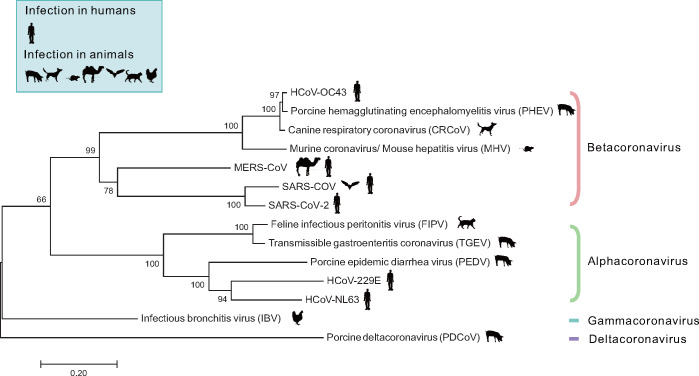
Phylogenetic tree of CoVs. The CoVs characterized here involve 4 genera with 14 species and classified into groups according whether it can infect human or not. Evolutionary distances of CoVs were calculated by RNA-dependent RNA polymerase (RdRp) sequences. Phylogenetic analyses were conducted by the maximum likelihood method in MEGA7. The scale bar indicates evolutionary distance in substitutions per site. Numbers next to the branches indicate the score of each clade based on bootstrap test (1000 replicates). The accession numbers of CoV sequences used for identification are SARS-CoV (NC_004718.3), SARS-CoV-2 (NC_045512.2), HCoV-229E (NC_002645.1), HCoV-OC43 (NC_006213.1), HCoV-NL63 (NC_005831.2), ERS-CoV (NC_019843.3), CRCoV (KX432213.1), FIPV (NC_002306.3), TGEV (NC_038861.1), IBV (NC_001451.1), MHV (AC_000192.1), PDCoV (KX022605.1), PEDV (NC_003436.1), and PHEV (KY994645.1).

According to phylogenetic relationships and genomic structures, CoVs can be classified into four genera: alphacoronavirus, betacoronavirus, gammacoronavirus, and deltacoronavirus. The genera and host types of CoVs discussed in this review are summarized in Figure 1. CoV infections are concentrated mainly to upper respiratory and gastrointestinal tract. According to specific virus and host cell types, the symptoms and pathological damages caused by CoVs are quite different. Some CoVs, like HCoV-NL63, HCoV-229E, and HCoV-OC43, continually circulate in human population and produce mild symptoms such as common cold, while severe acute respiratory syndrome coronavirus (SARS-CoV), middle east respiratory syndrome coronavirus (MERS-CoV), and SARS-CoV-2 produce severe respiratory illness with high morbidity and mortality (Cui et al., 2019; Ye et al., 2020). Strikingly, the outbreak of COVID-19 pandemic caused by SARS-CoV-2 has infected >14.7 million people in the world and killed >600 thousand people until the end of July 2020. In addition to affecting human health, some of the CoVs also seriously threaten the animal husbandry, as summarized in Table 1. In this review, we only focus on the CoVs that have been reported to interact with host cytoskeletons.

**Table 1:** Abbreviations of viruses.

Type	Abbreviation	Full name
Infection in humans	HCoV-229E	Human coronavirus 229E
	HCoV-OC43	Human coronavirus OC43
	HCoV-NL63	Human coronavirus NL63
	MERS-CoV	Middle East respiratory syndrome coronavirus
	SARS-CoV	Severe acute respiratory syndrome coronavirus
	SARS-CoV-2	Severe acute respiratory syndrome coronavirus-2
Infection in animals	CRCoV	Canine respiratory coronavirus
	FIPV	Feline infectious peritonitis virus
	IBV	Infectious bronchitis virus
	MHV	Murine coronavirus/ mouse hepatitis virus
	PDCoV	Porcine deltacoronavirus
	PEDV	Porcine epidemic diarrhea virus
	PHEV	Swine/porcine hemagglutinating encephalomyelitis virus
	TGEV	Transmissible gastroenteritis coronavirus

Cytoskeleton is an intricate network in eukaryotic cells, which comprises three major types of cytoskeletal polymers including actin filaments (AFs), microtubules (MTs), and intermediate filaments (IFs), allowing cells to perform multiple functions in a united way, such as connecting to the external environment, coordinating forces to move and change shapes, transporting vesicles through the cytoplasm, and spatially organizing the contents (Fletcher and Mullins, 2010; Wickstead and Gull, 2011).

AFs are most abundant polymers for large number of cells. Actin exists in monomeric form as globular actin or G-actin and in filamentous form called F-actin or microfilaments (Döhner and Sodeik, 2005). Quick assembly and disassembly are regulated by a variety of actin-binding proteins, which enable AFs to provide mechanical support, determining cell shape, migration, and division (Pollard and Borisy, 2003). Importantly, AFs can construct sheet-like extensions such as lamellipodia, membrane ruffles, and blebs, finger-like protrusions like microvilli and filopodia, or dot-like podosomes (Taylor et al., 2011). The actin cortex beneath the plasma membrane can be a barrier for virus entry or egress (Marsh and Bron, 1997). Moreover, with the help of motor protein myosin, AFs can serve as tracks for short-range transport of cargoes.

MTs are long, hollow cylindrical polar structures with dynamic plus-end and minus-end, assembled by heterodimers of α- and β-tubulin (Desai and Mitchison, 1997; Downing, 2000; Döhner and Sodeik, 2005). The minus-ends of MTs are often attached to the sites where MTs are nucleated, and the most essential activity is to form different types of microtubule-organizing centers (MTOCs), whereas plus-ends are pointing to plasma membrane, which contributes to the intracellular transportation of MT-bound vesicles (Wu and Akhmanova, 2017; Akhmanova and Steinmetz, 2019). The coexistence of assembly and disassembly at the ends generates dynamic and unstable characteristics of MTs. Importantly, MTs combine with motor protein families to take part in long-distance transport in neuronal dendrites and axons (Prokop, 2013). MTs are also key components of respiratory cilia. Thus, the homeostasis of MTs is closely related to neurological and respiratory diseases.

Among these filaments, IFs are distinguished by the medium size (∼10 nm-diameter) compared to AFs (∼7 nm) and MTs (∼24 nm) (Franke et al., 1978). Over 70 proteins encode IFs and their expressions vary with cells and tissues. Based on the structure and sequence composition, IFs proteins are classified into six types, including keratins (types I and II); desmin, glial fibrillary acidic protein (GFAP), and vimentin (type III); neurofilaments, nestin, and α-internexin (type IV); nuclear lamins (type V); and filensin and phakinin (type VI or others) (Herrmann et al., 2007; Lowery et al., 2015; Yoon and Leube, 2019). Unlike AFs and MTs, IFs are more stable, usually surround the nucleus, and extend throughout the cytoplasm, serving as scaffolds and participating in intracellular organization, membrane trafficking, and signaling transduction (Lowery et al., 2015). More and more studies demonstrate that abnormalities of IFs lead to severe pathogenesis like epithelial to mesenchymal transition (EMT) and neuronal diseases (Liem and Messing, 2009; Rout-Pitt et al., 2018).

Over the years, extensive studies have discovered that most viruses hijack cytoskeleton network to fulfill their own infection (Foo and Chee, 2015; Denes et al., 2018; Miranda-Saksena et al., 2018; Bedi and Ono, 2019; Zhang et al., 2019), which inspires a wide variety of exciting research avenues. In this review, we outline the important roles of AFs, MTs, and IFs in the life cycle of CoVs and analyze the relationship between host cytoskeleton and CoVs-induced pathological changes.

## Roles of AFs in CoV infection

### Entry

**Figure 2:**
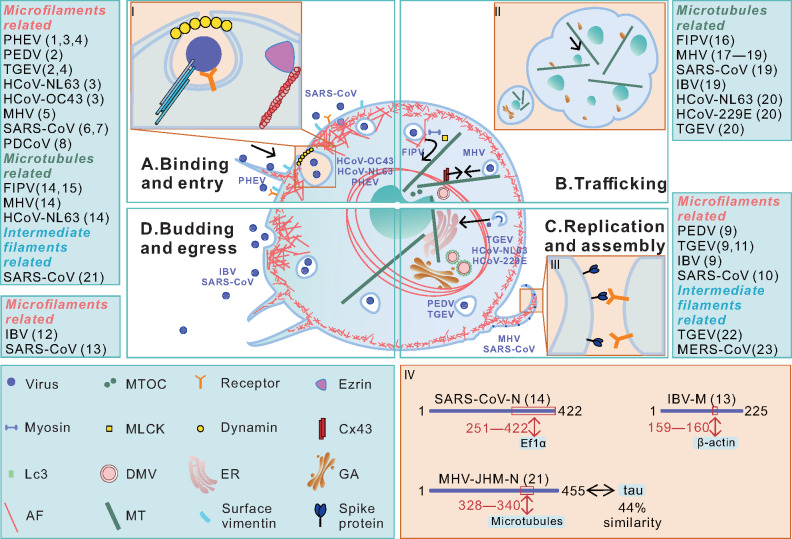
Multi-functional roles of host cytoskeleton in the life cycle of CoV. The solid line boxes dividing a host cell into four parts refer to different phases during CoV infection. The numbers in brackets correspond to the references in Tables 2‒4. (**A**) The role of cytoskeleton in the binding and entry process of CoVs. SARS-CoV binds to the specific host receptor where IFs participate as the co-receptor. Subsequently, PHEV surfs along filopodia to reach the appropriate entry area. The internalization of HCoV-OC43, HCoV-NL63, and PHEV, like endocytosis, is accompanied by dynamic cortical actin rearrangements. Ezrin inhibits the entry and fusion of SARS-CoV but promotes PDCoV infection, and dynamin participates in the endocytic process under some circumstances (I). (**B**) The role of cytoskeleton in CoV trafficking. MTs guide the trafficking of internalized vesicles containing FIPV from plasma membrane to replication sites. MHV infection restricts MT-mediated Cx43 delivery to cell membrane via the interaction between N protein and tubulins. MTs guide the translocation of fragmented GA into the center of the syncytia during MHC infection (II). (**C**) The role of cytoskeleton in replication and assembly of CoVs. MHV and SARS-CoV cause cell membrane ruffling, extensive filopodia, and the formation of macropinocytosis in the late stage of infection. At cell surface, S protein mediates fusion events with neighboring cells (III). The juxtanuclear ring formed by AFs supports PEDV or TGEV genome replication and protein synthesis. TGEV, HCoV-NL63, and HCoV-229E components rely on MTs for transport in ERGIC. The specific amino acid sequences of viral protein interact with the cytoskeleton and related protein (IV). (**D**) Actin polymerization contributes to IBV and SARS-CoV budding and egress.

**Figure 3:**
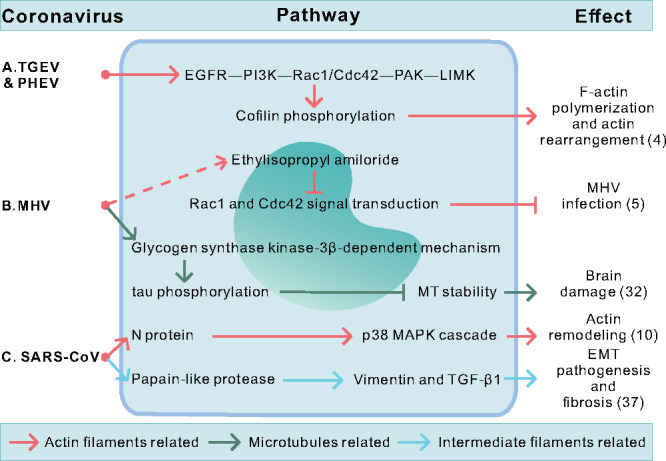
Summary of cytoskeleton-related signal transduction in CoV infection. Five pathways involving three viruses are summarized. The numbers in brackets correspond to the references in Tables 2‒5. (**A**) Early in the infection, TGEV and PHEV cause the phosphorylation of cofilin by signal transduction to further regulate the AF network. (**B**) MHV infection changes the AF and MT-related signaling pathways, involving several small GTPase and kinases, to complete viral infection and aggravate pathological damage. (**C**) SARS-CoV proteins result in actin remodeling, EMT pathogenesis, and fibrosis by regulating respective signaling pathways.

After binding to the target cell, viruses must migrate to favorable sites for entry, usually by virus surfing (Figure 2A). A study using porcine hemagglutinating encephalomyelitis virus (PHEV) labeled with the lipophilic fluorescent dye indodicarbocyanine (DiD) revealed that bound viruses surfed toward the foot of filopodia via actin retrograde flow at ∼30 min postinfection. During this process, AFs depolymerized and transient blebs were formed on the cell surface (Li et al., 2017). Similar results were also observed in porcine epidemic diarrhea virus (PEDV)- and transmissible gastroenteritis coronavirus (TGEV)-infected IPEC-J2 cells. After viruses reached the entry site, AFs retracted and concentrated around plasma membrane. Then actin bundles lined with plasma membrane for virus internalization (Zhao et al., 2014). Pharmacological stabilization of actin cortex by jasplakinolide prevented HCoV-OC43, HCoV-NL63, and PHEV from penetrating host cells and resulted in retention of virions on actin cortex or unstructured actin deposits, suggesting that CoV internalization requires dynamic actin rearrangements (Li et al., 2017; Milewska et al., 2018; Owczarek et al., 2018). Besides, viruses also take advantage of cytoskeleton-regulating signaling pathways as part of their infection processes (Figure 3).

Previous studies have shown that TGEV and PHEV could induce cofilin phosphorylation by activating cellular EGFR‒PI3K‒Rac1/Cdc42‒PAK‒LIMK signaling pathway at the early stage of infection, thereby causing F-actin polymerization and rearrangement and further promoting virus entry (Figure 3A; Hu et al., 2016; Lv et al., 2019). Blocking Rac1 and Cdc42 signal transduction by ethylisopropyl amiloride inhibits murine coronavirus (MHV) infection (Figure 3B; Koivusalo et al., 2010). These results suggest that dynamic actin cytoskeleton and relevant signaling pathways strongly contribute to virus entry.

Nevertheless, membrane‒actin linker ezrin could interact with SARS-CoV S endodomain and hinder virus entry and fusion (Figure 2A). Knockdown of ezrin or expression of dominant-negative (DN) ezrin increases virus entry (Millet et al., 2012; Millet and Nal, 2015). Further analysis identified that the F1 lobe of ezrin FERM domain, the last 8 C-terminal residues, and the membrane-proximal cysteine cluster of SARS-CoV S endodomain are responsible for this interaction (Millet et al., 2012). Another study found that N protein of porcine deltacoronavirus (PDCoV) upregulated ezrin, which may further facilitate viral infection by manipulating the host cytoskeleton network and cell signaling (Lee and Lee, 2015). These results suggest that AFs cooperate with other host proteins to play dual roles during the entry of CoVs.

### Replication and assembly

After entry, AFs further undergo rearrangement during CoV replication and assembly. In PEDV- or TGEV-infected IPEC-J2 cells, AFs retract from plasma membrane, form a juxtanuclear rings, and bind to virus particles near nuclear membrane (Figure 2C), supporting viral genome replication and viral protein synthesis. Disruption of AF dynamics by jasplakinolide or cytochalasin D blocked actin ring formation and inhibited replication and release of PEDV, TGEV, and infectious bronchitis virus (IBV) (Surjit et al., 2004; Gov and Gopinathan, 2006; Wang et al., 2009; Zhao et al., 2014; Sun et al., 2017). AF reorganization can be induced by viral protein expression and related signaling pathway. SARS-CoV N protein induces p38 mitogen-activated protein kinase (MAPK) cascade, which plays an important role in actin remodeling (Figure 3C; Surjit et al., 2004; Gerits et al., 2007; Zhao et al., 2014). Moreover, actin-binding protein filamin A interacts with TGEV S protein, which is essential for the retention of S protein at the ERGIC (Trincone and Schwegmann-Weßels, 2015).

### Egress

The budding and egress of CoVs are mainly related to AFs. Previous study found that the interaction between β-actin and M protein of IBV is essential for virus assembly and budding. Further analysis identified that amino acids A159 and K160 on M protein are important for this interaction (Figure 2C; Gov and Gopinathan, 2006; Wang et al., 2009). Moreover, a study using atomic force microscope and scanning electron microscopy has shown that SARS-CoV infection resulted in proliferation of pseudopodia and thickening of AFs below the subcellular surface at the late stage of infection, which may provide the bending force to extrude the virus particles (Figure 2D; Ng et al., 2004). Together, these results indicate that actin network involves in assembly and expelling of the progeny CoV particles probably by providing additional force for membrane bending. Roles of AFs and related proteins at different stages of CoVs life cycle are summarized in Table 2.

**Table 2:** Summary of the roles of AFs in CoV infection.

Phase	Virus (genera)	Description	References	No.
Entry	PHEV (β)	Bound virus surfs toward the foot of filopodia	Li et al. (2017)	(1)
	PEDV (α), TGEV (α)	AFs line with plasma membrane for virus internalization	Zhao et al. (2014)	(2)
	HCoV-NL63 (α), HCoV-OC43 (β), PHEV (β)	Virus internalization requires dynamic actin rearrangements	Li et al. (2017); Milewska et al. (2018); Owczarek et al. (2018)	(3)
	TGEV (α), PHEV (β)	Virus hijacks actin-regulating signaling pathways to promote entry	Hu et al. (2016); Lv et al. (2019)	(4)
	MHV (β)	Blocking Rac1 and Cdc42 signal transduction inhibits virus infection	Koivusalo et al. (2010)	(5)
	SARS-CoV (β)	Knockdown of ezrin or expression of DN ezrin increases virus entry	Millet et al. (2012); Millet and Nal (2015)	(6)
		Ezrin interacts with SARS-CoV S endodomain	Millet et al., 2012	(7)
	PDCoV (δ)	N protein of virus upregulates ezrin	Lee and Lee, 2015	(8)
Replication and assembly	PEDV (α), TGEV (α), IBV (γ)	Actin rings support viral genome replication and viral protein synthesis	Surjit et al. (2004); Gov and Gopinathan (2006); Wang et al. (2009); Zhao et al. (2014); Sun et al. (2017)	(9)
	SARS-CoV (β)	N protein induces p38 MAPK cascade and remodel actin	Surjit et al. (2004); Gerits et al. (2007); Zhao et al. (2014)	(10)
	TGEV (α)	The interaction of filamin A with S protein is essential for the retention of S protein at the ERGIC	Trincone and Schwegmann-Weßels (2015)	(11)
Egress	IBV (γ)	The interaction between β-actin and M protein is essential for virus assembly and budding	Gov and Gopinathan (2006); Wang et al. (2009)	(12)
	SARS-CoV (β)	Infection results in proliferation of pseudopodia and thickening of AFs at the late stage of infection	Ng et al. (2004)	(13)

## Roles of MTs in CoV infection

### Entry

Dynamin, an MT-related protein, is responsible for the endocytic process of feline infectious peritonitis virus (FIPV), MHV, and HCoV-NL63 infections (Figure 2A), and dynamin inhibitory peptide, siRNA of dynamin, and DN dynamin all effectively block virus internalization (Van Hamme et al., 2008; Burkard et al. 2014; Milewska et al., 2018). However, the internalization of viral protein‒antibody complexes in FIPV-infected monocytes did not require Rho-GTPases, actin, or dynamin, which is contrary to FIPV internalization (Dewerchin et al., 2008; Van Hamme et al., 2008). These results indicate the complexity of MTs-associated CoV entering processes.

### Transport

Once entering host cell, CoVs-containing vesicles run along MTs to move from the plasma membrane toward replication sites (Figure 2B). By visualizing the endocytosis process during FIPV infection, it was found that internalized vesicles were associated with MTs just 1 min after initial internalization. After 10 min, CoVs-containing vesicles reached MTOC. Chemical stabilization or depolymerization of MTs cannot block endocytosis but keep the vesicles close to the plasma membrane, instead of being transported to the cell center (Dewerchin et al., 2014). These results suggest that MTs are critical in guiding the transportation of internalized CoVs-containing vesicles.

CoVs can be transported from the ER to Golgi apparatus (GA) for assembly, which is in an MT-dependent manner (Figure 2C). Double-membrane vesicles (DMVs) that associate with RTCs are considered as the CoV replication site, whereas expression of SARS-CoV NSP6 can also induce single-membrane vesicles surrounding MTOC (Hagemeijer et al., 2010; Angelini et al., 2013). MT-associated protein 1 light chain 3 (LC3) could act as the cross-node of multiple pathways to take part in the formation process of DMV during the infection of MHV, SARS-CoV, and IBV (Prentice et al., 2004a, b; Cottam et al., 2011; Reggiori et al., 2011; Maier et al., 2013). Nonstructural protein 2 (NSP2) of MHV is recruited to RTCs by virtue of its C terminus and associated with DMV cytoplasmic side. Moreover, the work using live-cell imaging demonstrated that NSP2 moves through the cytoplasm in an MT-dependent manner. Nocodazole-induced depolymerization of MTs cannot affect the formation of RTCs but causes scattered distribution of RTCs in the cytoplasm, instead of concentrating in the perinuclear region, and reduced titer of MHV (Hagemeijer et al., 2010; Biswas and Das Sarma, 2014). S and M proteins have been proved to interact with tubulin during the infection of several alphacoronaviruses, such as TGEV, HCoV-NL63, and HCoV-229E, either directly or indirectly. MT depolymerization changes the distribution of these proteins. There are less S proteins incorporated into virions, while M proteins remain unaffected. Moreover, MTs promote the replication efficiency of TGEV, and MT depolymerization does not completely inhibit its infection. Therefore, this conservative strategy of MT-dependent CoV replication is at least one potential competent avenue (Rüdiger et al., 2016).

The viral evolutionary homolog can mimic fundamental cell process for the sake of the viral life cycle. For instance, residues 328‒340 of neurotropic murine coronavirus (JHMV) N protein were found to be aligned optimally with MT-binding domain of tau, where overall 20% identity and 42% similarity were uncovered. The amino acid sequence homology between N protein and tau provides a possible molecular mechanism for the interaction between viral protein and MTs (Pasick et al., 1994; Kalicharran and Dales, 1995). Roles of MTs at different stages of CoV life cycle are summarized in Table 3.

**Table 3:** Summary of the roles of MTs in CoV infection.

Phase	Virus (genera)	Description	References	No.
Entry	FIPV (α), MHV (β), HCoV-NL63 (α)	Inhibition of dynamin effectively blocks virus internalization	Van Hamme et al. (2008); Burkard et al. (2014); Milewska et al. (2018)	(14)
	FIPV (α)	Internalization of virus does not require Rho-GTPases, actin, or dynamin	Dewerchin et al. (2008); Van Hamme et al. (2008)	(15)
Transport	FIPV (α)	MTs guide the transportation of internalized virus-vesicles.	Dewerchin et al. (2014)	(16)
	MHV (β)	Depolymerization of MTs cannot affect the formation of RTCs, but causes scattered distribution of RTCs	Hagemeijer et al. (2010); Biswas and Das Sarma (2014)	(17)
		The specific interaction between tau and JHMV N protein	Pasick et al. (1994); Kalicharran and Dales (1995)	(18)
	MHV (β), SARS-CoV (β), IBV (γ)	LC3 acts as the cross-node of multiple pathways to take part in the formation process of DMVs	Prentice et al. (2004a); Prentice et al. (2004b); Cottam et al. (2011); Reggiori et al. (2011); Maier et al. (2013)	(19)
	HCoV-NL63 (α), HCoV-229E (α), TGEV (α)	S and M proteins have been proved to interact with tubulin during the infection	Rüdiger et al. (2016)	(20)

## Roles of IFs in CoV infection

The replication cycle of CoVs is initiated by the binding of S protein to cell surface receptors (Fung and Liu, 2019). Intriguingly, vimentin IFs could act as the co-receptor to participate in the process of virus entry. For instance, SARS-CoV infection increases the expression of vimentin, and cell surface vimentin cooperates with angiotensin-converting enzyme 2 (ACE2) to construct the receptor for SARS-CoV S protein. Anti-vimentin antibody successfully blocked virus entering Vero E6 cells and its neutralizing efficiency was close to that of anti-ACE2 antibody, indicating that vimentin has the potential to be a target for antiviral therapies (Figure 2A; Yu et al., 2016). In addition, vimentin can bind to N protein of TGEV, and knockdown of vimentin significantly decreased cell-associated virus, suggesting that vimentin plays an essential role in CoV replication (Zhang et al., 2015). Furthermore, cytokeratin 18 (CK18)-expressing epithelial cells are the prevailing target of MERS-CoV, rather than CK5/6 or CK14-expressing cells, indicating that various types of IFs are related to cell tropism of CoVs (Haverkamp et al., 2018). Specific events in which IFs participate are listed according to the genera of viruses as well as the phases during infection (Table 4).

**Table 4:** Summary of the roles of IFs and multi-cytoskeleton networks in CoV infection.

Phase	Virus (genera)	Description	References	No.
Entry	SARS-CoV (β)	Cellular surface vimentin as the co-receptor for S protein.	Yu et al. (2016)	(21)
Replication	TGEV (α)	Vimentin binds to viral N protein, which is essential for viral replication	Zhang et al. (2015)	(22)
	MERS-CoV (β)	CK18-expressing epithelial cells are the prevailing target cell	Haverkamp et al. (2018)	(23)
Multi-cytoskeleton	FIPV (α)	AF-related proteins and MTs participate in the intracellular trafficking of internalized vesicles	Dewerchin et al. (2014)	(24)
	PHEV (β)	The propagation of virus depends on MTs and IFs in the nerve cell	Hara et al. (2009)	(25)
	TGEV (α)	Dynamin 2 assists with actin to participate in the internalization of virus	Wang et al. (2020)	(26)
		Several cytoskeleton-related proteins express differentially	Zhang et al. (2013)	(27)
	IBV (γ)	Numerous cytoskeletal and related proteins associate with virion	Emmott et al. (2010); Cao et al. (2012); Dent et al. (2015)	(28)

## Crosstalk among multi-cytoskeleton networks in CoV infection

CoVs could utilize comprehensively three cytoskeleton networks to complete viral transport process. Transport from/to the cell periphery for short-range route is mediated by actin and its motor proteins like myosin, while long-range transport is mediated by MTs and the motor proteins dynein and kinesin (DePina and Langford, 1999; Langford, 2002; Dewerchin et al., 2014; Robinson et al., 2017). In FIPV-infected monocytes, small actin tails, myosin light chain kinase (MLCK), and myosin 1 cooperate with MTs to participate in the intracellular trafficking of internalized vesicles, which may be conducive to switch tracks from AFs to MTs (Figure 2B; Dewerchin et al., 2014). The propagation of swine hemagglutinating encephalomyelitis virus depends on MTs and IFs in neurons, which facilitate virus to be transported along the neuron cell body and axonal terminals (Hara et al., 2009). Moreover, dynamin 2 assists with actin to participate in the internalization of TGEV witnessed by single-virus tracking (Wang et al., 2020).

Cytoskeleton components were found to be candidates emerged from several CoV infection-related screens. Two-dimensional difference gel electrophoresis (2D DIGE) coupled with matrix-assisted laser desorption/ionization time-of-flight tandem mass spectrometry (MALDI-TOF-TOF/MS) identified 33 differentially expressed proteins in TGEV-infected swine testes cells. Surprisingly, 35.3% of them are cytoskeleton-related proteins such as β-actin, α-tubulin, keratin 19, and vimentin (Zhang et al., 2013). Another study identified numerous cytoskeletal and related proteins that associate with IBV virion, including tubulin α1 chain, tubulin β3 chain, tubulin β4 chain, tubulin β7 chain, vimentin, myosin-9, annexin A2, and actin α cardiac muscle 1 (Dent et al., 2015). Moreover, IBV infection upregulates the expression of vimentin and actin (Emmott et al., 2010). The proteomic analysis also found that the abundance of α-tropomyosin and vimentin increases with the virulence of IBV strains (Cao et al., 2012). Together, multi-cytoskeleton components involved in CoV infection are listed in Table 4, and these results indicate that cytoskeleton networks are tightly associated to CoV infection.

## CoV-related pathology involved in host cytoskeleton

### Cytokinesis

CoV infection can change the normal cytokinesis by affecting AFs. Elongation factor 1-α (EF1α) interacts with F-actin and promotes F-actin bundling, which is essential for the formation of contractile ring during cytokinesis (Yang et al., 1990; Kurasawa et al., 1996; Numata et al., 2000; Gross and Kinzy, 2005). Using yeast two-hybrid screen, it has been identified that the C terminus (amino acids 251‒422) of SARS-CoV N protein interacts with EF1α and induces aggregation of EF1α, which destroys the bundling of F-actin, thereby inhibiting protein translation and cytokinesis (Figure 2C; Zhou et al., 2008).

### Syncytia

AFs and MTs participate in the formation of syncytia induced by CoV infection. For example, MHV and SARS-CoV infections induce macropinocytosis, accompanied by membrane ruffling and extensive filopodia, which can facilitate S protein‒receptor interactions with neighboring cells and thereby is important for virus replication and cell‒cell fusion (Freeman et al., 2014; Figure 2C). In addition, MTs participate in the translocation of fragmented GA during CoV infection. Previous study has shown that GA was fragmented and translocated to the center of the syncytia, while MTs were rearranged and radiated toward syncytia in MHV infection, suggesting that MTs perhaps provide guidance for the transportation of GA into the center of the syncytia (Figure 2B; Kalicharran and Dales, 1996; Lavi et al., 1996).

### Brain damage and cilia loss

Several studies have shown that disruption of MTs is related to neurodegenerative diseases. For instance, MHV infection induces tau phosphorylation via glycogen synthase kinase-3β-dependent mechanism, which disrupts MT stabilizing capacity and thereby causes brain damage (Figure 3B; Kalicharran and Dales, 1995; Sy et al., 2011; Barbier et al., 2019). The progression of demyelinating disease is correlated with MT-dependent transport. Gap junctions (GJs) formed by connexin 43 (Cx43) and Cx47 are important for maintenance of central nervous system (CNS) homeostasis. MHV-A59 infection restricted MT-mediated Cx43 delivery to cell membrane via the interaction between MHV N protein and tubulins. Besides, MHV-A59 infection downregulated Cx47 expression, which resulted in GJ loss and further caused demyelination (Figure 2B; Basu et al., 2017). Interestingly, chemical disruption of MTs with colchicine and vinblastine significantly inhibits S protein-mediated neuronal transport and subsequent spread of RSA59 (MHV demyelinating strain) whereas RSMHV2 (MHV nondemyelinating strain) remains unaffected. This indicates that RSA59 uses MTs as a conduit for trans-neuronal spread. The difference between these two MHV strains in causing demyelination and axonal loss is determined by the dependence on MTs (Das Sarma et al., 2008, 2009; Biswas and Das Sarma, 2014).

Structural damage to the respiratory epithelium and abnormal ciliary function are the typical pathologic symptoms of CoV infection. Cilia is a composite structure based on MTs and presents on the cell surface (Soares et al., 2019). Several studies have found that CoVs with severe respiratory damage such as SARS-CoV, MERS-CoV, IBV, and canine respiratory coronavirus (CRCoV) cause cilia loss in the upper respiratory tract and lung, whereas low toxic HCoV-OC43 does not affect cilia functions (Chilvers et al., 2001; Nicholls et al., 2003; Villarreal et al., 2007; Priestnall et al., 2009; Mitchell et al., 2013; Essaidi-Laziosi et al., 2018; Haverkamp et al., 2018). These results suggest that the structure of MTs is associated with different pathogenesis of respiratory CoVs, which associates with cilia formation.

### Others

Troponin is a regulator of muscle tissue contraction, attached to the tropomyosin on AFs (Lehman et al., 2009). The level of troponin in the heart muscle of patients was increased during MERS-CoV and SARS-CoV-2 infections (Alhogbani, 2016; Chen et al., 2020; Guo et al., 2020; Inciardi et al., 2020; Lippi et al., 2020; Madjid et al., 2020; Shi et al., 2020). Importantly, patients with severe myocardial damage accompanied by high troponin levels have the higher risk of death (He et al., 2020).

IFs are also involved in certain cytopathic processes during CoV infection. SARS-CoV papain-like protease induces vimentin IF upregulation and activation of profibrotic cytokines TGF-β1, which results in EMT pathogenesis and fibrosis (Figure 3C; Li et al., 2012). Moreover, FIPV infection induces high expression of vimentin and mild expression of GFAP in astrocytes with severe inflammatory and necrotic changes, despite that vimentin is normally absent in CNS areas (Poli et al., 1997; Mesquita et al., 2016; Ziółkowska et al., 2017). These results indicate that vimentin expression could reflect a reactive or degenerative change of astrocytes. Furthermore, modifications in the phosphorylation state of neurofilaments are associated with multiple sclerosis during HCoV-OC43 infection (Tsunoda and Fujinami, 2002; Brison et al., 2014). CoV-related pathological events involved in the host cytoskeleton are listed in Table 5.

**Table 5:** Summary of CoV-related pathology involved in host cytoskeleton.

Phase	Virus (genera)	Description	References	No.
Cytokinesis	SARS-CoV (β)	The interaction between viral N protein and EF1α destroys AFs bundling and inhibits cytokinesis	Zhou et al. (2008)	(29)
Syncytia	MHV (β), SARS-CoV (β)	Infections induce micropinocytosis that can facilitate S protein‒receptor interactions with neighboring cells	Freeman et al. (2014)	(30)
	MHV (β)	MTs perhaps provide guidance for the transportation of GA into the center of the syncytia	Kalicharran and Dales (1996); Lavi et al. (1996)	(31)
Brain damage and cilia loss	MHV (β)	Infection induces tau phosphorylation and disrupts MT stabilizing capacity, thereby causing brain damage	Kalicharran and Dales (1995); Sy et al. (2011); Barbier et al. (2019)	(32)
		Infection restricts MT-mediated Cx43 delivery to the cell membrane via the interaction between N protein and tubulins	Basu et al. (2017)	(33)
		Chemical disruption of MTs significantly inhibits S protein-mediated neuronal transport and subsequent spread of RSA59 whereas RSMHV2 remains unaffected.	Das Sarma et al. (2008); Das Sarma et al. (2009); Biswas and Das Sarma (2014)	(34)
	SARS-CoV (β), MERS-CoV (β), HCoV-OC43 (β), CRCoV (β), IBV (γ)	Viruses cause cilia loss in the upper respiratory tract and lung, whereas low toxicity HCoV-OC43 does not affect cilia functions	Chilvers et al. (2001); Nicholls et al. (2003); Villarreal et al. (2007); Priestnall et al. (2009); Mitchell et al. (2013); Essaidi-Laziosi et al. (2018); Haverkamp et al. (2018)	(35)
Others	MERS-CoV (β), SARS-CoV-2 (β)	The level of troponin in the heart muscle of patients is increased in infection	Alhogbani (2016); Chen et al. (2020); Guo et al. (2020); Inciardi et al. (2020); Lippi et al. (2020); Madjid et al. (2020); Shi et al. (2020)	(36)
	SARS-CoV (β)	Papain-like protease induces vimentin upregulation and activation of TGF-β1	Li et al. (2012)	(37)
	FIPV (α)	Infection induces high expression of vimentin and mild expression of GFAP in astrocytes	Poli et al. (1997); Mesquita et al. (2016); Ziółkowska et al. (2017)	(38)
	HCoV-OC43 (β)	Modifications in the phosphorylation state of neurofilaments are associated with multiple sclerosis during infection	Tsunoda and Fujinami (2002); Brison et al. (2014)	(39)

## Perspective

Here, we summarize and highlight that three cytoskeletons AFs, MTs, and IFs are heavily involved in the life cycle and pathological damages caused by CoVs. Regulations between specific viral proteins and cytoskeleton-related proteins were focused and summarized in Table 6. As we are gaining a greater understanding on the regulation of cytoskeleton components and corresponding elaborate subcellular structures in the process of CoV infections, there are numbers of exciting and substantial questions worth future pursuing.

**Table 6:** Summary of the regulations between coronaviral proteins and cytoskeletal components.

Genera	Virus	Viral protein	Description	Cytoskeletal Components	Experimental approaches	References
α	TGEV	S	Interacts	AFs—filamin A	GST pulldown; IF	Trincone and Schwegmann-Weßels (2015)
		N	Interacts	IFs—vimentin	GST pulldown; co-IP; IF	Zhang et al. (2015)
	HCoV-NL63, HCoV-229E, TGEV	S	Interacts	MTs—tubulin	GFP Traps pulldown; MS; IF	Rüdiger et al. (2016)
β	SARS-CoV	S	Interacts	AFs—ezrin	Yeast two-hybrid screen; GST pulldown; siRNA; IF	Millet et al. (2012); Millet and Nal (2015)
			Interacts	IFs—vimentin	IP; extracellular chemical cross-linking; MS; IF	Yu et al. (2016)
		N	Interacts	AFs—EF1α	Yeast two-hybrid screen; IP; IF	Zhou et al. (2008)
		papain-like protease	Upregulates	IFs—vimentin	Proteomic analysis; western blotting; qRT-PCR assay	Li et al. (2012)
	MHV-JHMV	N	Homologous with	MTs—tau	Chemical inhibitors; electron microscopy; IF	Pasick et al. (1994)
	MHV-A59	N	Interacts	MTs—tubulins	IF; co-IP; animal models; frozen sections	Basu et al. (2017)
γ	IBV	M	Interacts	AFs—β-actin	Yeast two-hybrid screen; co-IP; IF; chemical inhibitors	Gov and Gopinathan (2006); Wang et al. (2009)
δ	PDCoV	N	Upregulates	AFs—ezrin	IF; fluorescence-activated cell sorting analysis; two-dimensional gel electrophoresis; peptide mass fingerprinting	Lee and Lee (2015)

IF, immunofluorescence assay; Co-IP, co-immunoprecipitation assay; MS, mass spectrometry; IP, immunoprecipitation assay.

- Since CoVs need to overcome barriers formed by AFs to successfully enter into or egress from host cells, it would be important to figure out how CoVs manipulate AFs and relevant binding proteins to regulate the curvature formation of host plasma membrane.
- It will be interesting to study how internalized CoVs switch transportation tracks from AFs to MTs.
- Considering that the neurodevelopmental disorders and respiratory tract damage caused by CoVs are MT-dependent, it is thus of great interest to study why and how CoVs disrupt the homeostasis of MTs in infected cells.
- Another key question is to understand the roles of different IF proteins in various host cells during CoV infection. Particularly, since vimentin could act as the co-receptor to be involved in the entry of SARS-CoV, it will be essential to investigate whether IF proteins function universally as a potential coronaviral (co)-receptor.

There is a substantial increase in our understanding of how host cytoskeleton network regulates CoV infection. Thorough exploration is imperative and starting now to provide new insights into cytoskeleton during CoV infections, most interestingly, from the perspective of cell biology. Therefore, a global understanding of host cytoskeleton during CoV infection will help to inspire new strategies to control infection and relieve CoV-related pathological damage.

## Funding

This study was supported by Shanghai Municipal Science and Technology Major Project (20431900402 and 2019SHZDZX02), the National Natural Science Foundation of China (31970660), Natural Science Foundation of Shanghai (19ZR1463000), Chemical Reagent & Instrumental Development Foundation of Shanghai (1914200700), ‘100 Talents Program’ from the Chinese Academy of Sciences, and Shanghai Talent Development Funding.

**Conflicts of interest:** none declared.
